# A Steered Molecular Dynamics Study of Binding and Translocation Processes in the GABA Transporter

**DOI:** 10.1371/journal.pone.0039360

**Published:** 2012-06-21

**Authors:** Søren Skovstrup, Laurent David, Olivier Taboureau, Flemming Steen Jørgensen

**Affiliations:** 1 Department of Drug Design and Pharmacology, Faculty of Health and Medical Sciences, University of Copenhagen, Copenhagen, Denmark; 2 Department of Computational Chemistry, H. Lundbeck A/S, Valby, Denmark; 3 Center for Biological Sequence Analysis, Department of Systems Biology, Technical University of Denmark, Building 208, Lyngby, Denmark; Universität Erlangen-Nürnberg, Germany

## Abstract

The entire substrate translocation pathway in the human GABA transporter (GAT-1) was explored for the endogenous substrate GABA and the anti-convulsive drug tiagabine. Following a steered molecular dynamics (SMD) approach, in which a harmonic restraining potential is applied to the ligand, dissociation and re-association of ligands were simulated revealing events leading to substrate (GABA) translocation and inhibitor (tiagabine) mechanism of action. We succeeded in turning the transporter from the outward facing occluded to the open-to-out conformation, and also to reorient the transporter to the open-to-in conformation. The simulations are validated by literature data and provide a substrate pathway fingerprint in terms of which, how, and in which sequence specific residues are interacted with. They reveal the essential functional roles of specific residues, e.g. the role of charged residues in the extracellular vestibule including two lysines (K76 (TM1) and K448 (TM10)) and a TM6-triad (D281, E283, and D287) in attracting and relocating substrates towards the secondary/interim substrate-binding site (S2). Likewise, E101 is highlighted as essential for the relocation of the substrate from the primary substrate-binding site (S1) towards the cytoplasm.

## Introduction

The anti-convulsive agent tiagabine is the only approved drug that works by inhibiting the gamma-aminobutyric acid (GABA) transporters (GATs) [1], namely GAT-1. The exact mechanism and site of inhibition of tiagabine in GAT-1 is, however, still unclear thereby limiting the development of new selective GAT inhibitors.

Detailed studies on the interactions between the GATs and their substrates and inhibitors were until recently hampered by the lack of a three-dimensional structure. In 2005 the first crystal structure of a homologues protein, the bacterial leucine transporter (LeuT) was released [2], revealing 12 transmembrane (TM) helices, two sodium ions, and a substrate, L-leucine, situated in the centre of the protein. The structure revealed a so-called outward-facing occluded state, with the primary substrate binding site (S1) being occluded, shielded from the extracellular solute by two gating residues, Y108 and F253, and on top of these a water-mediated salt bridge formed by R30 and D404. The latter salt bridge was later recognized as an important interim binding site [3] or even as a secondary allosteric substrate binding site (S2) [4]. However, the exact role of this site is still being debated [5]. Subsequently released crystal structures of LeuT reported outward-facing occluded conformations in complex with various ligands, and a single crystal structure revealed an open-to-out conformation with a small-molecule inhibitor, L-tryptophan, occupying the S1 binding site [6], [7], [8], [9], [10]. Based on these crystal structures, diverse computational simulations were performed to explore the translocation of LeuT [4], [11], [12], [13], [14].

In recent years, much effort has been focused on developing non-GAT-1 selective inhibitors to prevent degradation of the neurotransmitter [15], [16], [17], [18]. Unfortunately, a rational structure-based approach has not been possible due to the lack of a three-dimensional structure of a GAT. Recently, we published a carefully constructed three-dimensional homology model of GAT-1 using the LeuT crystal structures as templates [19]. With our GAT-1 homology model and our models of the other three GAT subtypes constructed following the same protocol, it is now possible to study ligand interactions *in silico*, to explore subtype differences and to ease the quest for subtype selective inhibitors. Further insight into the binding mechanism, i.e. insight into the association and translocation mechanism of substrates might give a clue to important subtype differences located outside the primary substrate binding site. Likewise, the mechanism of inhibition of drug transporters from this family is still not fully understood. For example, tricyclic antidepressants (TCAs) and selective serotonin reuptake inhibitors (SSRIs), both types being inhibitors of the serotonin transporter (SERT), were shown to bind in the extracellular vestibule in the crystal structures of LeuT [6], [7], [10], but in mutational studies on SERT their binding site was distinctly indicated to overlap with the binding site of the substrate, i.e. the S1 site [20], [21]. In the case of the GATs the small substrate-analog inhibitors generally behave as competitive inhibitors, but with the larger lipophilic derivatives there is no clear picture about their mode of action/inhibition. Thus tiagabine behaves as a competitive and mixed-type inhibitor dependent on the conditions [1], and other analogs show different inhibition kinetics with the GAT subtypes, and behave as either competitive, non-competitive, uncompetitive, or mixed-type inhibitors [22].

**Figure 1:**
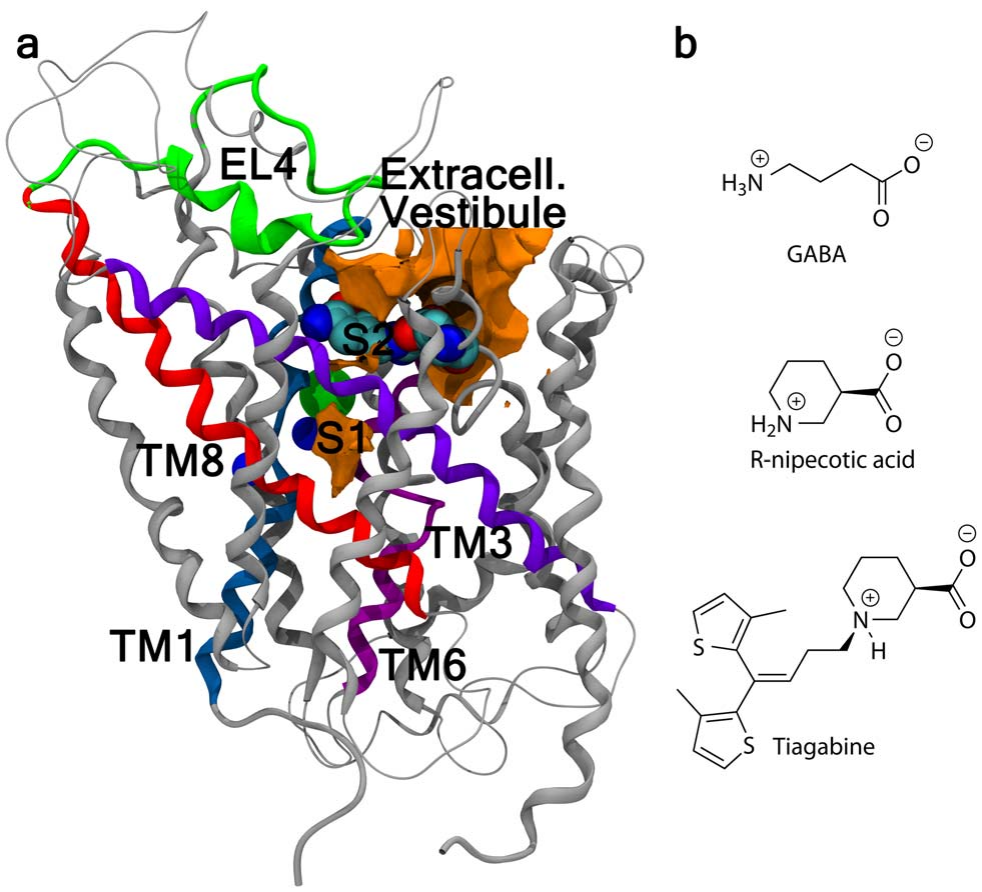
GAT-1 and GAT-1 ligand structures. (a) Three-dimensional representation of the homology model of the human GABA transporter GAT-1. Structural features mentioned in the text are marked. S1, the substrate binding site; S2, the secondary/interim binding site; TM1 (TM3, TM6 and TM8), transmembrane region 1 (3, 6 and 8); and EL4, extracellular loop 4. (b) Structures of the endogenous ligand gamma-aminobutyric acid, GABA, *R*-nipecotic acid, and the anti-convulsive drug tiagabine.

Here we present extensive steered molecular dynamics (SMD) simulations to study the substrate translocation pathway of human GAT-1. The validity of SMD simulations was first checked for the streptavidin-biotin complex by Grubmuller et al. [23] and this method has in recent years been applied to study a wide range of biological systems and issues (for a review see for example Sotomayor & Schulten [24] or Genchev et al. [25]) though primarily related to ligand (un)binding and/or related events, e.g. allosteric signaling (see for example Amaro et al. [26], Jensen et al. [27], or Mai et al. [28]). Likewise, the association and translocation pathway of LeuT was probed by the SMD approach [4], [11]. Here, we extend a published study on the binding mode of key substrates, GABA and *R*-nipecotic acid (cf. Figure 1) to GAT-1. By SMD we simulate dissociation- and association events of the two substrates in the outward-facing conformation of the transporter. We complete the translocation pathway by also steering GABA to the cytoplasmic side, thereby gaining insight into the full substrate translocation pathway and the underlying mechanisms involved in substrate translocation with emphasis on ligand-protein interactions. Finally, we address the riddle of the association mechanism of tiagabine (cf. Figure 1). Events are described phenomenologically and are compared and related to structural knowledge from literature data.

## Results

### Dissociation of GABA

**Figure 2:**
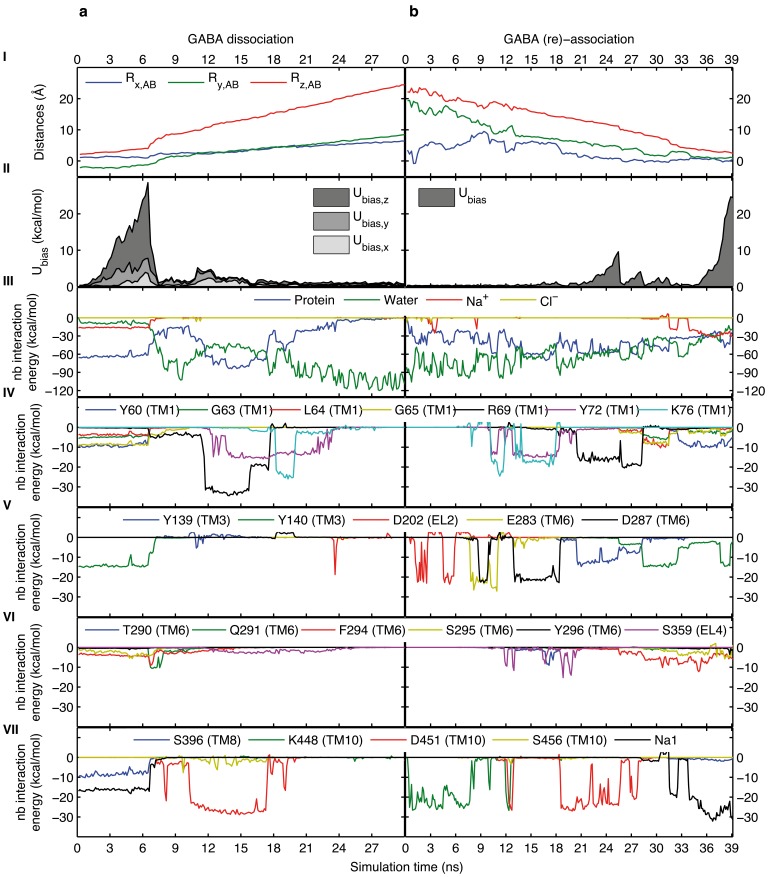
Dissociation and re-association of GABA. SMD profiles for GABA dissociation using displacement restraints (a), and GABA re-association using distance restraints (b). For both (a) and (b) the individual figures from top to bottom show the displacement of GABA relative to the center of mass (COM) of the chemical system (I); the biasing potential energy profile (II); non-bonded interaction energy profiles between GABA and the protein, water, sodium ions, and chloride ions (III); non-bonded interaction energy profiles between GABA and residues interacted with during simulation (IV-VII). Figures on the left-hand side share the same time-axis as does figures on the right-hand side. Note, for the displacement restrained simulation depicted in (A) the displacement vector is specified by its components, hence the biasing potential energy is resolved into its x-, y-, and z-components, while the biasing potential energy is given for the vector in space in the distance restrained simulation in (B). Generally, changes in the interaction pattern between GABA and the protein are reflected in e.g. the biasing potential energy profile and vice versa. Most notably is the departure from the primary binding site, S1. During the initial 6 ns of the dissociation simulation (left-hand side) GABA is firmly keeping the initial binding mode (i.e. interactions with particularly Y60, G65, Y140, S396 and Na1), while the biasing potential energy (II) is accumulating. After circa 6 ns the direct ionic or hydrogen bonding interactions to the protein and sodium ion (Na1) are solvated by water molecules (III) thereby facilitating GABA to depart from the binding site, which is visualized by a sudden change in the COM movements depicted in (I) and in the drop in the biasing potential energy in (II). At the S2 site the interactions between GABA and the protein are reinforced due to particularly the interactions to R69 and D451.

We previously reported the binding mode of GABA to the human GABA transporter [19]. In this binding mode, GABA is located in the S1 site, stabilized by an intramolecular interaction between the carboxylate and the amine, and by an ionic interaction with Na1 and hydrogen bonding interactions with Y60(O), G65(NH), Y140(OH), and S396(OH). This binding mode was used as input structure for the dissociation simulation of GABA, and steering was done using displacement restraints, at 0.179, 0.353, and 0.75 Å/ns in the x-, y-, and z-directions, respectively, applying a 5 kcal/mol·Å^2^ biasing force constant in each direction. Key details of the simulation are collected in the left-hand side of Figure 2 in terms of I) traces of the center of mass (COM) of GABA, II) evolvements of the biasing potential energy, III) non-bonded interactions between GABA and the protein, water, sodium and chloride ions, IV-VII) non-bonded interactions between GABA and residues interacted with along the translocation pathway (for clarity only residues interacting with an energy higher than 5 kcal/mol have been included).

Initially the extracellular vestibule was visually scrutinized to find the least hindered linear pathway from S1 through the extracellular vestibule to the extracellular side (cf. Figure 1). The large loop, EL4, situated in the extracellular vestibule is thought to function as a lid controlling which (and when) ligands enter the protein. It has been shown to be involved in ligand discrimination and the conformational mobility of the transporter [7], [29], [30], [31], [32], [33]. Following a route leading directly from S1, through S2, to the extracellular side, the small substrate molecules seem able to move through the extracellular vestibule without colliding with EL4. Thus, routes following this path were initially tested (data not included) and from these a nearly unhindered route was clearly visualized and employed in the simulation described below.

Throughout the initial 6.5 ns, GABA is tightly bound to the S1 site in the pose previously described experiencing circa 65 kcal/mol in non-bonded interaction energies to the protein and additional 20 kcal/mol to Na1 (Figure 2a, III). After 6.5 ns, and the accumulation of almost 30 kcal/mol in biasing potential energy (Figure 2a, II), interactions between GABA and S1 site residues (Y60, S396 etc.) are one-by-one ruptured and the ligand starts leaving the binding site (Figure 2a, IV-VII). First, the ligand amine interactions to Y60(O) and S396(OH) are replaced by interactions to Y140(OH) and F294(O), and GABA becomes sandwiched between the aromatic rings of Y140 and F294. The aliphatic GABA chain is forming hydrophobic contacts to the R69 side chain (at S2). The interaction between the ligand carboxylate and Y140(OH) is replaced with a contact to Q291(NH_2_), and the contact to Na1 is consequently solvated by a water molecule. After 7 ns GABA leaves the S1 site and is almost completely solvated by a shell of water molecules, though still tightly bound in a network of water mediated intramolecular and electrostatic interactions with residues from the S1 site (including Na1) and the S2 site. After 8 ns GABA is increasingly coordinated by S2 site residues, thus a water molecule is coordinated between Y139(OH), D451(CO_2_^−^) and the ligand amine, and after 10.2 ns D451 forms direct and persistent ionic contacts to the ligand amine. The side chain of R69 is first lining with the aliphatic GABA chain, thereby obstructing the drift of the ligand leading to a small build-up of the biasing potential energy (Figure 2a, II), and after 11.5 ns R69 rearranges to form ionic interactions with the GABA carboxylate via the guanidinium. While following the target movements, GABA remains directly bound to the S2 site residues for around 6 ns, where after the ligand carboxylate is transferred to Y72, located one helical turn above R69 in TM1, and then K76, located one turn above Y72. The interaction between the ligand amine and D451 is gradually solvated after 17.4 ns, and after almost 20 ns the GABA carboxylate is fully solvated. Though still situated in the extracellular vestibule, in an opening between TM1, TM6, TM10, TM11, and EL2 and EL4a, GABA is highly solvated by at least one shell of water molecules while it is following the target movements out of the vestibule. A video showing the SMD trajectory for the dissociation (and re-association, see below) of GABA is included as Video S1.

The dissociation simulation was repeated using a distance restraining protocol, defining the center of the intracellular gate as the counterweight to which the distance to GABA in the S1 site is increased during the simulation. In this way GABA is pushed (rather than being pulled as in the previous setting) from the S1 site towards the extracellular solute in the least biased setup possible by this kind of SMD approach. The simulation is summarized in Figure S1 and S2, which correspond to Figure 2a, I-III and Figure 2a, III-VII, respectively. In Figure S3 the potential (blue curve) and kinetic energy (red curve) of the system is followed throughout the simulation, and the applied force (blue curve) and the work (green curve) is followed as a function of the distance travelled by the ligand in the left-hand side of Figure S4.

Generally, the pathway and interaction patterns of this simulation are very similar to the ones observed from the displacement restrained simulation outlined above. In this second simulation less potential energy is accumulated to escape from the S1 binding site, and the biasing energy profile (Figure S1, II) generally shows fewer accumulations in accordance with the re-association described below (and sketched in Figure 2b, II). The ligand is following a very similar pathway from the S1 site through the S2 site. Through the extracellular vestibule to the extracellular solute a more comprehensive interaction pattern is sketched (compare Figure S1 and S2 from time  = 21ns to Figure 2a from time  = 18 ns). Thus, from the S2 site toward the extracellular solute GABA is following interaction sites at TM1 and TM6 (i.e. Y72, K76, D287, E283, D281). This interaction pattern is very much in agreement with the observations from the re-association simulation described below and supports the proposed pathway in the extracellular halve of the transporter.

During dissociation of the ligand no significant domain movements are observed (except for various loops which quite expectedly continuously changes conformation). The kinetic energy of the system (Figure S3, red curve) is stable throughout the simulation, while the potential energy is slightly decreasing between time 6 ns and 20 ns (Figure S3, blue curve). During this time interval GABA is leaving the S1 site and moving through the S2 site to the extracellular vestibule where it does not experience any further hindrance from the protein. Besides, the system is not completely in equilibrium (as it is an SMD simulation) and the energy of the system is therefore not expected to be completely stable. Likewise, the applied force and the work done on the system are displayed in Figure S4. From Figure S4 the work done on the system is accumulating during the initial approximately 21 Å, corresponding to the distance between the S1 site and the tip of the EL4 loop which GABA passes right after it has passed through the S2 site. S1 site residues are generally keeping or re-taking the initial (occluded state) conformation after GABA has left the site, except from the two lid residues, Y140 and F294, which are actually keeping a more open conformation in accordance with (but not identical to) the conformation observed in the crystal structure of the open-to-out conformation of LeuT [8].

### Dissociation of R-nipecotic Acid

The substrate dissociation simulation was repeated with *R*-nipecotic acid, an equipotent GABA-analogue acting as both a substrate and inhibitor of GAT-1. The trajectory from this simulation is visualized in Figure S5 in terms of graphs following I) ligand COM movements and evolvements of the biasing potential energy, II) non-bonded interactions between *R*-nipecotic acid and the protein, water, sodium- and chloride ions, III) non-bonded interactions between *R*-nipecotic acid and specific residues interacted with along the translocation pathway.

The trajectories of the dissociation of GABA and *R*-nipecotic acid show very similar events. Not only do they follow the same pathway, but they also show very similar force profiles and interaction profiles. There is, however, one interesting difference between the unbinding events of the two substrates; compared to GABA *R*-nipecotic acid is much more dependent on water molecules assisting in breaking the interactions to the protein in the initial binding mode in the S1 pocket. GABA is extremely flexible and moves in a worm-like fashion, changing interactions from one hydrogen bonding site to another nearby site. With *R*-nipecotic acid water enters the binding site and inserts in the interactions between the amine and the protein (i.e. the interactions with S396(OH) and Y60(O)) leading to a lower energy barrier for changing binding mode. Consequently, he piperidine can easily rotate and form new hydrogen bonding interactions to the opposite side of the binding site. In this way, the much more rigid ligand structure of *R*-nipecotic acid is compensated for by the involvement of water molecules in the interactions with the protein. Both ligands are, however, strictly dependent on water molecules to break the ionic interaction to Na1.

### Association of GABA

To support the extracellular substrate pathway outlined above, the GABA dissociation simulation was continued in the opposite direction thereby steering the ligand back towards the substrate binding pocket. For the (re)-association simulation a distance restraining protocol was applied, with a biasing velocity of 0.75 Å/ns and a biasing force constant on 10 kcal/mol·Å^2^. With a distance restraining protocol ligand steering is less biased and the ligand therefore responds better to favorable interaction sites, and accordingly the re-association pathway may not be identical to the dissociation pathway.

**Figure 3:**
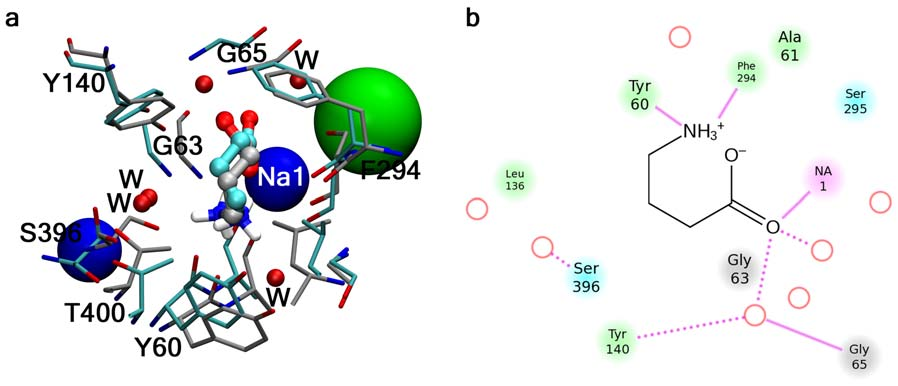
GABA binding upon re-association. (a) Comparison of the initial binding mode of GABA (from which the dissociation simulation was started) and the binding mode obtained upon re-association to the S1 binding site. The initial binding mode is color-coded with grey carbon atoms, and the binding mode obtained by SMD is color-coded with cyan carbon atoms. Red spheres represent water molecules involved in GABA binding upon re-association (see text), the blue spheres represent the two structural sodium ions, and the green sphere represents the structural chloride ion. Nitrogen and oxygen atoms are color-coded blue and red, respectively. (b) 2D ligand interaction diagram sketching the binding mode obtained from the association simulation (prepared via 2D ligand interaction available through Maestro [55]. Red circles represents water molecules involved in GABA binding, a dashed line represents a hydrogen bond to a protein side chain while a solid line represents an interaction with a protein backbone atom. Protein residues are color-coded as: green – hydrophobic, blue – polar, gray – glycine, pink – metal.

The ligand COM traces from the association simulation (Figure 2b, I) clearly show greater mobility of the ligand in the extracellular vestibule (initial 13 ns) compared to the first dissociation simulation (Figure 2a, I) and in full agreement with the secondly described (less biased) dissociation simulation (Figure S1,I). Likewise, lower potential energy barriers are observed during association than in the initially described dissociation simulation (cf. Figure 2a, II and 2b, II), and in full agreement with the secondly described dissociation simulation. Early in the association simulation GABA forms ionic interactions with K448(NH_3_^+^) and the flexible EL2 loop closes down on GABA and interacts via D202(CO_2_^−^). Being situated at the entrance to the extracellular vestibule GABA moves to the opposite side of the vestibule forming interactions to the TM6 residues E283 and D287, and K76 in TM1. In this way, positioned between TM1 and TM6 GABA approaches the S2 site and follows largely the same route to the S1 site as was found in the dissociation simulation. After almost 36 ns simulation GABA ends up in the S1 site with both GABA and the surrounding residues adopting essentially the same binding conformation as in the starting structure of the dissociation simulation, although water molecules are not depleted from the binding site and are therefore also interacting with the ligand. In Figure 3 the binding mode obtained upon (re)-association of GABA is compared to the previously proposed binding mode (by docking) from which the dissociation simulation was started.

It is encouraging that three simulations (two dissociation and one association) with different setups reveal essentially identical substrate pathways and interaction patterns in the extracellular half of the protein. The fact that GABA ends up in a binding mode essentially identical to the initial binding mode, though including also few interacting water molecules, while also the protein conformation is preserved, is supportive of both the extracellular substrate pathway but also the previously hypothesized binding mode. Figure 2a, IV-VII and 2b, IV-VII reveal that the non-bonded interactions between the ligand and the protein are on the same order of magnitude at the S2 and the S1 sites. In addition Na1, which is located in the S1 site, is forming ionic interactions to GABA in the order of 20–40 kcal/mol, and thus functions as an attractive force driving the ligand from the S2 site to the S1 site. A video showing the SMD trajectory for the dissociation and re-association of GABA is included as Video S1.

### GABA Translocation

**Figure 4:**
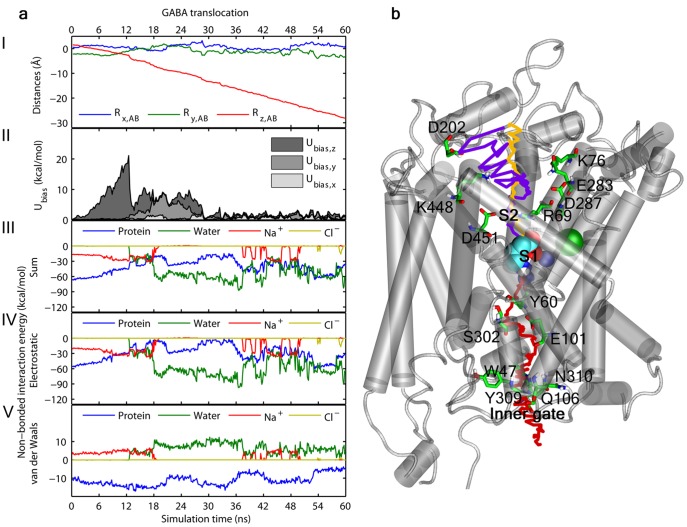
Translocation of GABA. (a) SMD profiles for the translocation of GABA using displacement restraints. The individual figures from top to bottom show: the distance of GABA relative to the center of mass (COM) of the chemical system (I); the biasing potential energy profile (II); the sum of the non-bonded interaction energies between GABA and the protein, water, sodium- and chloride ions, respectively (III); the electrostatic contribution to the non-bonded interaction energy profiles between GABA and the protein, water, sodium- and chloride ions (IV); the van der Waals contribution to the non-bonded interaction energy profiles between GABA and the protein, water, sodium- and chloride ions (V). Compared to the dissociation simulation in Figure 2 GABA is following the target movements (I) to a larger extend in the beginning of the simulation (i.e. while leaving the binding site) though still accumulating significant biasing potential energy (II) until after circa 12 ns water is entering the binding site and disrupts the interaction to particularly the sodium ion, Na1, (III-V). The displacement of GABA from the binding site to the cytoplasmic gate is associated with a continuous slight accumulation of biasing potential energy, which is expected because of the associated reorientation of the protein from the occluded outward-facing to the open-to-in conformation of the protein. It is worth noting that while the non-bonded electrostatic interactions between GABA and the protein are continuously decreasing until GABA reaches the cytoplasmic gate, and particularly until it is leaving the binding site, the non-bonded vdW interactions are actually slightly increasing until the departure from the binding site, and overall relatively stable until GABA reaches the cytoplasmic gate. (b) Traces of the substrate translocation pathways from the extracellular dissociation of GABA using displacement restraints (yellow), extracellular re-association of GABA using distance restraints (purple), and translocation to the cytoplasm using displacement restraints (red). GABA is shown in the S1 site in cyan spheres, and important residues interacted with during simulation are shown in green sticks.

In order to determine the complete pathway for the translocation of substrates across the membrane, we also simulated the translocation of GABA to the cytoplasm. Thus, GABA was pulled from the S1 site to the cytoplasmic side via displacement restraints, starting from the same binding mode in the occluded outward-facing protein conformation as the dissociation simulations described above. A second GABA molecule was placed in the S2 site in accordance with the allosteric role of this site proposed by Shi et al. [4], though the role of this site as a secondary allosteric binding site or an interim binding site has been disputed [5]. Inclusion of a ligand molecule in the S2 site will provide a rationale of whether the S2 site most likely appears as an allosteric binding site or rather as an interim binding site, based on the stability of binding to this site. Visual inspections indicated that the cytoplasmic substrate pathway follow the z-axis. The velocities in the x- and y-direction were therefore set to 0 Å/ns with a low biasing force constant on 1 kcal/mol to weakly restrain the ligand movements to the z-direction only. The protein is expected to undergo significant conformational changes, and the pulling velocity in the z-direction was therefore only 0.5 Å/ns (compared to 0.75 Å/ns in the dissociation simulations) with a biasing force constant on 10 kcal/mol·Å^2^. In Figure 4a the SMD profiles for the GABA translocation process are collected.

**Figure 5:**
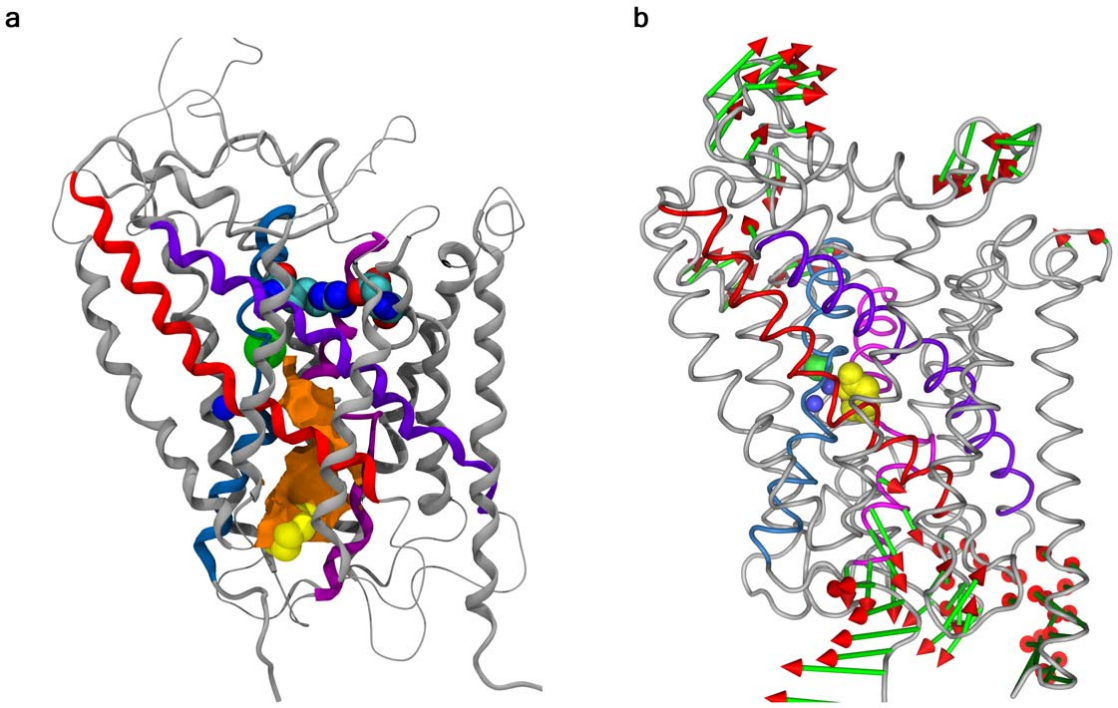
GAT-1 upon substrate translocation. (a) Cartoon representation of GAT-1 after 33 ns simulation with GABA (yellow spheres) located at the cytoplasmic gate. The orange surface shows the shape of the revealed channel leading from the S1 site to the cytoplasm. (b) Cartoon showing the backbone of GAT-1 in the initial occluded state conformation, with GABA (yellow spheres) located in the S1 site. The green and red arrows indicate the direction and size of the principal backbone movements observed during the translocation simulation (from the simulation applying displacement restraints) (This Figure was prepared with Pymol [59]. In both figures the four TM helices forming the S1 site are highlighted, i.e. TM1 (blue), TM3 (purple), TM6 (pink), and TM8 (red).

In accordance with the dissociation simulation GABA shows very stable binding to the S1 site during the first 8 ns, where after it slowly moves towards the gating residue, Y60. The ionic coupling to GABA causes Na1 to leave its binding site and follow GABA. Consequently, the unwound part of TM6 is slightly displaced. TM1 and TM8 move slightly apart and open up a narrow water channel in the vicinity of Y60 leading to the solvation of the GABA carboxylate and Na1. After 17.6 ns Y60 changes conformation and decisively breaks the interaction between GABA and Na1 as the tyrosine inserts between them. TM6 is slightly displaced through a change in the backbone dihedrals (*phi;psi*) of G301 (situated in the unwound part of TM6), from around (100°; −15°) to (−100°; 150°) creating a rather hydrophobic channel leading from the S1 site towards the cytoplasmic side. This channel is formed by the side chains of residues in TM1, TM2, and TM6. The carboxylate of E101 (located in TM2) contacts the GABA amine and assists GABA in moving from the S1 site into the newly formed channel. The channel is closed to the cytoplasmic side by a gate formed by F53, and in a second layer, polar residues from the N-terminal and IL3 (connecting TM6 and TM7), mainly W47, Q106, Y309, N310 and N314. Reaching F53 the biasing potential energy slightly accumulates until 32.5 ns when GABA moves amine-first in between the side chain of F53 and the aromatic system of W47. From here GABA moves relatively unhindered through the gate to the cytoplasmic side. Figure 5a shows GABA located at the cytoplasmic gate and the channel leading from the S1 site to the cytoplasmic site, and in Figure 5b the initial protein structure is shown and arrows indicate the location, direction and size of the major protein perturbations. The trace of the full trajectory pathway is shown in Figure 4b including key residues involved in ligand-protein interactions. A video showing the SMD trajectory for the translocation of GABA is included as Video S2.

During translocation to the cytoplasm the extracellular halve of the protein is slightly narrowing, while it is expanding in the cytoplasmic halve. The main determinant of the conformational perturbations is the cytoplasmic halve of TM6, which moves downward (toward the cytoplasm) and away from particularly TM1. Consequently, a tunnel leading from the S1 site to the cytoplasmic side is formed, and after the ligand has entered the tunnel the binding site is actually narrowed. This is illustrated in Figure S6 in which distances between the helices forming the primary binding site, i.e. TM1, TM3, TM6, and TM8, are monitored at the cytoplasmic and extracellular side, as well as at the S1 and S2 sites. The observed domain movements are directly related to ligand unbinding events, and are clearly reflected in the monitored distances. For example 1) at 9 ns Na1 is leaving its binding site leading to a destabilization/movement of particularly the unwound part of TM6, which is also transmitted to TM3; 2) at circa 18 ns a water channel is formed; 3) from 20 ns GABA is leaving the S1 site, and 4) at 35 ns GABA reaches the cytoplasmic gate. During and after the observed domain movements the secondary structure elements of the protein are largely conserved.

A similar SMD simulation, where leucine was pulled to the cytoplasmic side of the LeuT, was previously reported by Shi et al. and followed up on by Zhao et al. [4], [14] In the SMD simulation from this work the substrate, leucine, follows a pathway emerging between the cytoplasmic halves of TM1 and TM6, resulting in a relative down- and outward movement of particularly the inner halve of TM1. In the present SMD simulation of GAT-1, GABA is following the same translocation pathway, but instead of plain TM1 movements we observe a similar and distinct movement of particularly the cytoplasmic halve of TM6, downward and away from TM1. In Figure S7 the described domain movements (of particularly TM1 and TM6) are shown in a cartoon representation. For the purpose of comparison the representation in Figure S7 is mimicking the representation used by Zhao et al. [14].

The translocation simulation was repeated in a distance restrained setup, in which the center of the very extracellular entrance to the protein was defined as the counterweight to which the distance to the ligand in the S1 site is increased during the simulation. This setup is placing very little bias on the translocation pathway as the ligand is pushed away from its current position instead of being pulled toward a given point, and this is considered the least biasing setup possible by this algorithm. The trajectory is followed in Figure S8 in graphs following I) GABA COM movements, II) the biasing potential energy profile, III) non-bonded interactions between GABA and the protein, water, sodium and chloride ions, and in Figure S9 in terms of non-bonded interaction profiles between GABA and protein residues interacting with during translocation. Furthermore, the potential (blue curve) and kinetic energy (red curve) of the system is followed throughout the simulation in Figure S10, and in the right-hand side of Figure S4 the applied force (blue curve) and the work (green curve) is followed as a function of the distance travelled by the ligand.

The simulation is showing largely the same trends as in the displacement restrained simulation. GABA is, though, following a translocation route slightly closer to TM3. Consequently, instead of mainly TM6 movements, this translocation simulation shows equal TM1 and TM6 movements, and is therefore considered being in-between the displacement restrained simulation described above and that reported by Shi et al. in the LeuT [4].

Despite of the reported protein perturbations taking place during translocation, binding site residues are generally keeping or returning to the initial conformation after passage of the ligand. In comparison to the dissociation simulation the height of the potential energy barriers that the ligand has to pass to follow the target movements is of similar magnitude (compare the biasing potential energy profiles in Figure 2a/Figure S1 and Figure 4a/Figure S8 from the two dissociation simulations and the two translocation simulations). The same conclusion is drawn from the magnitude of the applied force in the dissociation and translocation simulation depicted in Figure S4 (blue curve). However, the green curves in Figure S4 reveal the work done on the system to add up being three times higher in the translocation simulation. This is of course expected as the protein is taking an outward-facing conformation from the beginning of both simulations and translocation therefore involves greater perturbations of the protein. From this the involvement of the structural sodium ions (and the chloride ion) as the driving forces for the release to the cytoplasmic site can be rationalized (that is, the energetically uphill transport of the neurotransmitter is driven be the energetically downhill co-transport of the sodium ions [34], [35]). Unfortunately, it was not possible to simulate this by the employed simulation setups, and the exact transport mechanism of the sodium (and chloride) ions remains to be fully elucidated.

The role of the S2 site substrate molecule during translocation of a substrate molecule from the S1 site was evaluated from the two translocation simulations outlined above (as well as from other initial simulations not reported here). The S2-site GABA molecule seems not important for the translocation mechanism, as it tends to diffuse away from the S2 site, or at least only keep the interaction to the arginine, R69. However, to completely rule out its role as an allosteric modulator more experimental evidence is needed as well as other simulation setups (e.g. MD simulations applying metadynamics to sample the energy landscape during translocation with and without a second substrate molecule occupying the S2 site).

### Inhibitor Binding, Tiagabine Association

Tiagabine, an analogue of *R*-nipecotic acid with a lipophilic diaromatic side chain attached to the amine, is a GAT-1 selective inhibitor, but unlike *R*-nipecotic acid it is not a substrate of the transporter. While *R*-nipecotic acid was hypothesized to bind in the bottom of the S1 site interacting with residues in TM1 and TM8, we hypothesized tiagabine to bind in the S1 site interacting with TM1 and TM6, with the lipophilic side chain placed in the rather hydrophobic area between the S1 and the S2 sites [19].

Initially, tiagabine was dissociated from the suggested binding mode in GAT-1 via SMD (data not included), the purpose being to lift the EL4 loop (residing in the extracellular vestibule) a few Angstrom to facilitate a less hindered association of tiagabine. Apart from the EL4 movement no significant domain movements were observed during the dissociation simulation. The structure was prepared for the association simulation by moving tiagabine by hand to the extracellular domain, above EL4, with no direct contacts to the protein. Subsequently, the system was relaxed through a short equilibration simulation.

**Figure 6:**
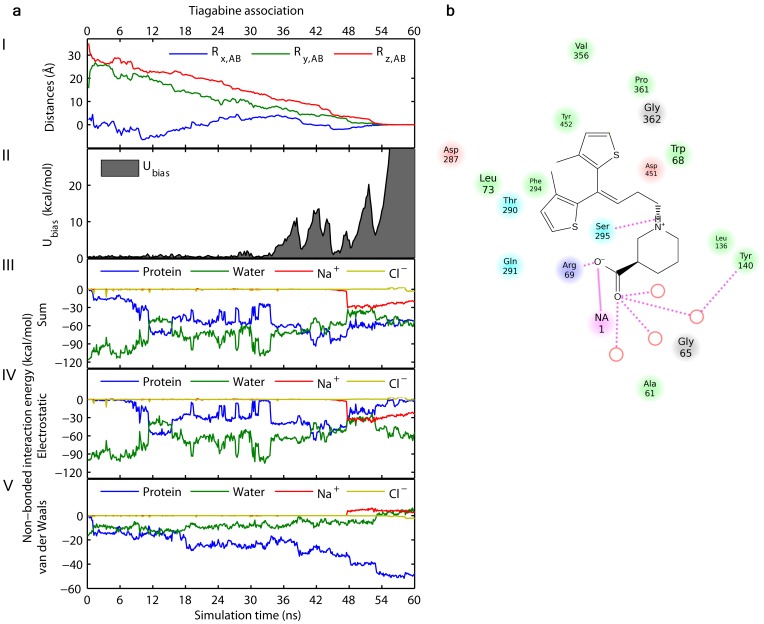
Tiagabine association. (a) Graphs following the trajectory of the association of tiagabine (using distance restraints). The individual figures from top to bottom show: the distance of tiagabine relative to the selected point in the bottom of the S1 binding site towards which tiagabine was pulled (I); the biasing potential energy profile (II); the sum of the non-bonded interaction energy profiles between tiagabine and the protein, water, sodium- and chloride ions (III); the electrostatic energy contribution to the non-bonded interaction energies between tiagabine and the protein, water, sodium- and chloride ions (IV); the van der Waals energy contribution to the non-bonded interaction energies between tiagabine and the protein, water, sodium- and chloride ions (V). Tiagabine is moving unhindered with the target movements through the extracellular vestibule until it binds to the S2 site after circa 34 ns. From this point the biasing potential energy is repeatedly accumulating though only by small amounts until tiagabine reaches the S1 site from where it cannot move any further into the protein. Compared to the biasing potential energy accumulated during translocation of GABA to the cytoplasm (Figure 4) the potential energy barriers for tiagabine reaching the S1 site are considerable lower than translocation of a substrate. (b) 2D ligand interaction diagram sketching the binding mode obtained from the association of tiagabine towards the S1 site in GAT-1. Residues are colored according to their properties: charged (pink or purple), polar (blue) and hydrophobic (green). Hydrogen bonds are shown as lines and water molecules as red open circles.

For the association simulation the nipecotic acid part of tiagabine was used as the steering selection, and steering was done via distance restraints. The biasing force profile is shown in Figure 6a, II together with traces of the x,- y,- and z-coordinates of the COM of the nipecotic acid part of tiagabine in Figure 6a, I. Figure 6a, III-V show the detailed non-bonded interactions. From the information in Figure 6a tiagabine interacts with the protein early in the simulation, but is moving unhindered following the steering forces until 34 ns. Indeed, early in the simulation tiagabine interacts with the protein through hydrophobic interactions as it buries the thiophene rings in EL6 and the extracellular part of TM12. The nipecotic acid part is fixed by water-mediated contacts to E202 (EL2) and K448 (TM10), which becomes a direct ionic interaction maintained for 19 ns. At that time tiagabine approaches the S2 site and the R69–D451 salt bridge, and at 33.6 ns the ligand carboxylate is inserted in the salt bridge via ionic contacts to R69, and a water mediated contact to D451. The biasing force slowly accumulates until the salt bridge is broken and tiagabine moves towards the S1 site. Doing so, the R69 side chain keeps the interaction to the tiagabine carboxylate and follows the movements towards the S1 site. With the R69 side chain sandwiched between tiagabine and TM1, two water molecules are trapped in a pocket between tiagabine, TM1, TM6, and the side chain of Q291. The biasing potential energy accumulates almost 15 kcal/mol and forces one of the water molecules to become inserted between the chloride and TM6, in the original position of the S295 side chain. Consequently, S295 and the TM6 backbone from F293 through the unwound part to L300 are displaced (up to 4.5 Å measured for the F294 C-alpha). This displacement does not show any violation of the backbone dihedral angles as examined via Ramachandran plots (data not shown). The displacement of the TM6 stretch slightly and widens the entrance to the binding site, and tiagabine moves into the S1 site. After 44 ns simulation R69 moves towards its usual conformation, and tiagabine is positioned in a binding mode very similar to the one suggested previously [19], with a hydrogen bonding contact between the amine and F294(O), an ionic interaction between the carboxylate and Na1, and a water mediated hydrogen bond between the carboxylate and Y140(OH). After 52.3 ns tiagabine moves deeper into the S1 site and the biasing potential starts accumulating dramatically as the unwound part of TM1 hinders any further advance of tiagabine. A video showing the SMD trajectory for the association and binding of tiagabine is included as Video S3.

Perturbations, like the one observed in the unwound part of TM6, must be examined with great care. However, in spite of the displacement of the unwound part of TM6, the simulation and the obtained binding mode are considered reliable for the following reasons. The simulation was repeated (results not included) with tiagabine showing a very similar association. We previously docked tiagabine via induced fit docking (IFD), obtaining a binding mode very similar to the one obtained by SMD. The poses from IFD were subjected to several long equilibrium MD simulations, revealing no significant domain movements. One of the MD simulations even showed the same mode of interaction with the Y140(OH), namely a water bridged hydrogen bonding interaction between the tiagabine carboxylate and the tyrosine. This mode of interaction was likewise observed in the crystal structure of LeuT with the small inhibitor tryptophan [8]. Furthermore, in the serotonin transporter (SERT) antidepressants were very convincingly pinpointed to share binding site with the substrates, namely the S1 site [20], [21].

## Discussion

We have presented SMD simulations covering the entire substrate translocation pathway in GAT-1, and we have also rationalized the association of the drug tiagabine inhibiting GAT-1. The present simulations together with substantial information from the literature enable us to present a qualified and detailed substrate translocation mechanism focusing on important ligand-protein interactions.

The role of the charged/polar residues at the extracellular entrance is to attract and guide the substrate into the extracellular vestibule. In the simulations this role was fulfilled by EL2 (particularly D202), EL4 (particularly A358 and S359), and EL6 (particularly Y527). EL4, EL5, and EL6 were shown to be important for ligand selectivity and affinity, and EL4 was also shown to change the degree of solvent exposure in response to conformational changes of the protein [29], [30], [31], [32], [36]. Thus, the extracellular loops are attracting and guiding the substrate/inhibitor into the extracellular vestibule. In the extracellular vestibule the ligand carboxylate is forming a strong interaction with the cationic side chain of either K76 or K448, which are located oppositely in the very extracellular part of TM1 and TM10, respectively. K448 was shown to be implicated in interactions with both substrates and antidepressants [31], [36], [37], and a K76Q mutant likewise showed some inhibition of transport [38]. Being bound to K76 the nearby residues in TM6, E283 and D287, assist in a tight binding and relocation of the ligand towards the S2 site. The D287G mutant experienced a reduction in substrate transport to circa 30% of wild type transporter [39]. D287 was also shown to be solvent exposed in the outward-facing protein conformation, while E283 was insensitive to the applied inhibition conditions [38]. The ligand is firmly relocated towards the S2 site, one helical turn at a time, to Y72 and then to R69. Y72 mutants showed significant decrease in transport activity/GABA uptake [40], [41].

At the S2 site R69 and D451 are anchoring the carboxylate and amine, respectively. R69 was shown to be essential for activity [41], [42], [43], and R69 and D451 were both participating in interactions with TCAs and SSRIs in GAT-1 and in LeuT [6], [7], [36]. (The SSRI and TCA binding site in SERT was, however, pinpointed to the S1 site, and not the S2 site as suggested by e.g. the LeuT crystal structures [20], [21]).

From S2 the substrate approaches the S1 site via a narrow rather hydrophobic channel, in which mainly W68 and Y139 are interacting with the ligand. Generally, W68 and Y139 mutants severely lowered the transporter functionality [41], [44], [45], [46], although the Y139W mutant actually increased transport activity [40]. The non-bonded interactions between the ligand and the protein are on the same order of magnitude at the S1 and the S2 sites. With Na1 (located at the S1 site) contributing by additional 20–40 kcal/mol in interaction energy when interacting with the ligand carboxylate, Na1 plays a crucial role as the driving force in attracting the ligand from S2 to S1. Entering the S1 site the ligand passes through the gating residues, Y140 and F294, which shields the S1 site to the extracellular media. Mutants of the two gating residues retained practically no transport activity [38], [40], and the LeuT crystal structures clearly demonstrate their role in closing the binding site and interacting with the ligand [2], [6], [7], [8], [9], [10].

With the substrate situated in the S1 site the transporter changes conformation to an inward-facing conformation, initially leading to an opening of a water-channel between TM1 (at Y60) and TM8. Water hydrates the ligand carboxylate and, with Y60 changing conformation, the contact to Na1 is broken and the tunnel to the cytoplasmic side opens. Y60 has also experimentally been demonstrated to be a key residue for selectivity [43], [47], [48]. E101 forms an ionic contact to the GABA amine and assists in pulling it from S1 into the cytoplasmic tunnel. At this position only a glutamate is tolerated, as even the chemically equivalent aspartate, E101D, maintained only 1% GABA transport, and no transport activity was detected with other mutants [39]. From the simulation it can be rationalized why an aspartate is not tolerated at this position, as the aspartate side chain is simply too short to reach the translocation pathway and interact with the ligand. From S1 to the cytoplasmic gate the interactions to the protein are largely of hydrophobic character or water mediated. The cytoplasmic gate is formed by N-terminal residues (primarily W47), TM1 residues (mainly F53), TM2 residues (mainly Q106), and IL3 residues (mainly Y309, N310, and N314). At the N-terminal R44 and W47 were shown to be essential, as only lysine was accepted in place of R44 (maintaining 15% activity), and only aromatic residues were tolerated replacing W47 [49], [50], [51]. Y309 (IL3) was likewise shown to be important as the Y309F mutant showed a reduced activity to approx. 25%, whereas both serine and tryptophan completely abolished transport activity [40]. These experimental observations are rationalized by our simulations by the observation, that Y309 is forming a T-shaped pi-stacking interaction with W47 and hydrogen binding interaction (via the hydroxyl group) to the D402 carboxylate, thus in this way Y309 is forming a very central piece of the intracellular gate. Finally, the N-terminal was indicated to form a gating domain with IL4 [52], which in our model is reproduced through particularly three ionic interaction-pairs, namely D43–R417, R44–D410, and D45–R420, which are kept until the protein is taking up the open-to-in conformation.

## Methods

We previously described the applied homology model of the human GABA transporter subtype 1 (GAT-1) in terms of the construction of the model, and the subsequent docking and MD simulations of key ligands, GABA, *R*-nipecotic acid, and tiagabine [19].

SMD simulations were set up and carried out using Desmond v. 2.2 [53], [54] compiled for Maestro [55] with the OPLS-AA 2005 force field, and the included biasing force plugin (documented in the Desmond User Guide).

Via *System Builder* the protein was embedded in a POPC lipid bilayer in an orthorhombic box of TIP3P waters and 0.15 M Na^+^ and Cl^−^ ions with 20 Å solvent/membrane buffer. The *solvate-pocket* script was used to fill water molecules in vacant sites in the protein and molecules placed between the protein and the membrane were manually removed. The system was energy-minimized and equilibrated via the equilibration protocol described previously [19] including also a 30 ns equilibrium MD simulation of the membrane and solvent phase in which the protein heavy atoms were restrained. Simulations were run at 310 K in the NPT ensemble with the Nose-Hoover thermostat and Martyna-Tobias-Klein barostat using anisotropic coupling. In SMD simulations weak restraints on the z-coordinate of selected C-alpha atoms in TM2, TM4, TM5, TM7, and TM9 prevented the protein in moving in the membrane in response to the exerted force, and x-y restraints on two C-alpha atoms (Y226 and S515), prevented the protein in floating in the box (this is only a visualization issue).

The biasing potential is applied as a time-dependent moving harmonic spring. With this approach, an external steering force is applied to selection *B* (the ligand), which is restrained with respect to a second selection, *A* (in the simulations presented here selection *A* is defined as the entire chemical system). The steering force is distributed and scaled by the mass of the atoms of the selection. The biasing potential was used in two forms, namely as displacement and distance restraints. That is, distance restraints are defined as a vector in space specified by a force constant, ***k***, and a velocity vector relative to the center of mass (COM) of selection *A*, and displacement restraints are essentially a vector in space defined by its components, force constant ***k (k_x_, k_y_, k_z_)*** and velocity vector ***v*** **(** ***v_x_, v_y_, v_z_*** **)**.

In dissociation and (re)-association simulations in the extracellular part of the protein steering velocities of 0.75 Å/ns (for distance restrained simulations) and 0.75 Å/ns in the z-direction (for displacement restrained simulations) were applied with 10 kcal/mol·Å^2^ force constants. In the distance restrained simulations the COM of the ligand was pulled towards Y60(O) situated in the bottom of the S1 binding site. It is important that the velocities of the target movement (i.e. the restraining position) are carefully balanced with the pulling forces. Traces of ligand COM movements (displayed as COM displacement vs. time) should show smooth traces with small plateaus, reflecting favorable interaction sites or obstructions of ligand displacement in the direction of the exerted force. This is in accordance with the requirements of e.g. Jarzynski’s equality [56], [57]. In the translocation simulations the protein is expected to undergo conformational changes, and consequently slightly slower velocities of 0.5 Å/ns were applied with 10 kcal/mol·Å^2^ force constants.

Unbinding events of both *R*-nipecotic acid and GABA were reevaluated and affirmed, thus dissociation of GABA was repeated with the same displacement restrained setup (not included here) as well as with displacement restraints using the intracellular gate as counterweight (selection *A*) as described in the text. Both simulations showed very similar dissociation events. Dissociation of nipecotic acid was repeated twice showing very similar trends. Simulations with *R*-nipecotic acid used to study the relationship between applied forces and pulling velocities (not included in this manuscript) confirmed the role of water assisting in releasing the ligand from the binding site in simulations applying low force constants, while simulations applying too large force constants showed an unphysical rupture.

Only one tiagabine isomer is included, although it is recognized that the configuration at the protonated amine might take two isoforms (and will continuously change between these in solution). Likewise, it is recognized that the thiophene rings are restrained to practically one conformation during simulation, although other conformations are also possible.

Specific residue atoms/groups are specified in parenthesis after the residue index, e.g. Y60(O) denotes the backbone oxygen of tyrosine 60, and S396(OH) denotes the side chain hydroxyl of S396. All graphs are smoothed over 25 frames/data points. For all graphs following non-bonded interaction energies between the ligand and specific residues only residues with interaction energies below −5 kcal/mol are displayed. Notice, these are interaction energies and does not take solvent effects, entropy etc. into account and are therefore not to be mistaken for relative or absolute binding energies or affinities. Trajectories were visualized and analyzed with VMD [58].

## Supporting Information

Figure S1 **GABA dissociation using distance restraints.** Graphs following the trajectory of the dissociation of GABA by distance restraints. The individual figures from top to bottom show: traces of the COM of GABA showing the distance of GABA relative to the cytoplasmic gate (I); the biasing potential energy profile (II); the non-bonded interaction energy profiles between GABA and the protein, water, sodium- and chloride ions, divided into the vdW and the electrostatic contributions as well as the sum of these (III).
(TIF)

Figure S2 **GABA dissociation using distance restraints.** Non-bonded interaction energy profiles between GABA and residues interacted with during simulation.
(TIF)

Figure S3 **Evolvement of the kinetic and potential energy of the chemical system during dissociation of GABA.** The kinetic energy (blue) is stable throughout the simulation, while the potential energy (red) is slightly decreasing between time 6 ns and 20 ns. During this time interval GABA is leaving the S1 site and is moving through the S2 site to the extracellular vestibule where it does not experience any further hindrance from the protein. The system is not in full equilibrium and hence is not the energies of the system. From the low-biased dissociation simulation with distance restraints.
(TIF)

Figure S4 **Applied force and work.** Comparison of the applied force (blue curve) and the accumulated work done on the system (green curve) as a function of the distance traveled by GABA for the low-biased distance restrained dissociation (left-hand side) and translocation (right-hand side) simulations. The graphs reveal that the height of the potential energy barriers to cross are of equal magnitude in the two simulations, but the work necessary to translocate to the cytoplasm is three times the work to dissociate to the extracellular media.
(TIF)

Figure S5 **SMD profile for the dissociation of** ***R-*** **nipecotic acid.** The individual figures from top to bottom show: the displacement (shown as lines) of the center of mass (COM) of *R-*nipecotic acid relative to the COM of the chemical system and the associated biasing potential energy profile (shown as stacked surfaces) (I); non-bonded interaction energy profiles between *R-*nipecotic acid and the protein, water, sodium- and chloride ions (II); non-bonded interaction energy profiles between *R-*nipecotic acid and residues interacted with during simulation (III).
(TIF)

Figure S6 **Intramolecular helix-helix distances from the GABA translocation simulation using displacement restraints.** Graphs following the distances between the helices forming the S1 substrate binding site: TM1, TM3, TM6, and TM8. At the cytoplasmic gate the helices clearly move, and particularly the inner half of TM6 moves downwards and away from TM1. At the extracellular side the distances are relatively stable.
(TIF)

Figure S7 **Structural changes associated with reorientation to the inward-open conformation.** The initial outward-facing occluded protein conformation (grey) is compared to the resulting inward-open conformation (orange) resulting from the translocation of GABA to the cytoplasm (using displacement restraints). GABA (shown as light-blue spheres) is located at the cytoplasmic gate. The helices forming the S1 binding site, TM1, TM3, TM6, and TM8, are marked. The illustration is comparable to the illustration by Zhao et al. of LeuT [14].
(TIF)

Figure S8 **GABA translocation to the cytoplasm using distance restraints.** The individual figures from top to bottom show: traces of the COM of GABA showing the distance of GABA relative to the extracellular entrance to the protein (I); the biasing potential energy profile (II); the non-bonded interaction energy profiles between GABA and the protein, water, sodium- and chloride ions, divided into the vdW and the electrostatic contributions as well as the sum of these (III).
(TIF)

Figure S9 **GABA translocation to the cytoplasm using distance restraints.** Non-bonded interaction energy profiles between GABA and residues interacted with during simulation.
(TIF)

Figure S10 **Evolvement of the kinetic and potential energy of the chemical system during translocation of GABA.** The kinetic energy (blue) is stable throughout the simulation, while the potential energy (red) is slightly decreasing. The system is not in full equilibrium and hence is not the energies of the system. From the low-biased translocation simulation with distance restraints.
(TIF)

Video S1 **SMD trajectory of dissociation (by displacement restraints) and re-association (by distance restraints) simulations of GABA from the S1 substrate binding site in the human GAT-1.**
(MPG)

Video S2 **SMD trajectory of the translocation (using displacement restraints) of GABA to the cytoplasm of the human GAT-1 from the occluded outward-facing protein conformation.**
(MPG)

Video S3 **SMD trajectory of the association and binding of tiagabine to human GAT-1.**
(MPG)
