# Supplementary figures and images for: A Steered Molecular Dynamics Study of Binding and Translocation Processes in the GABA Transporter
Source: PLoS One. 2012 Jun 21;7(6):e39360. doi: 10.1371/journal.pone.0039360 (PMC3380839; doi:10.1371/journal.pone.0039360)

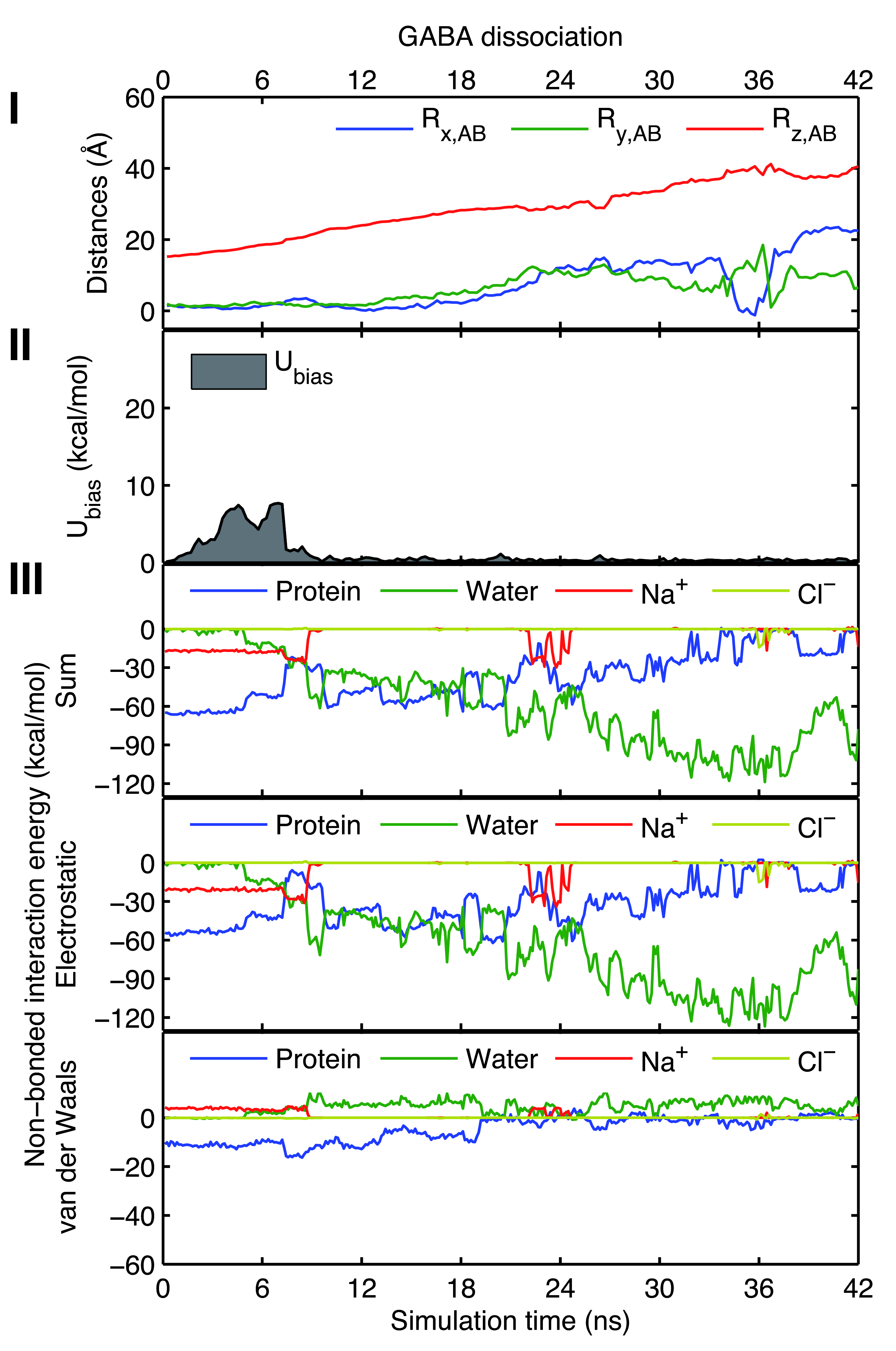

Supplement: Figure S1 — GABA dissociation using distance restraints. Graphs following the trajectory of the dissociation of GABA by distance restraints. The individual figures from top to bottom show: traces of the COM of GABA showing the distance of GABA relative to the cytoplasmic gate (I); the biasing potential energy profile (II); the non-bonded interaction energy profiles between GABA and the protein, water, sodium- and chloride ions, divided into the vdW and the electrostatic contributions as well as the sum of these (III). (TIF) [file pone.0039360.s001.tif]

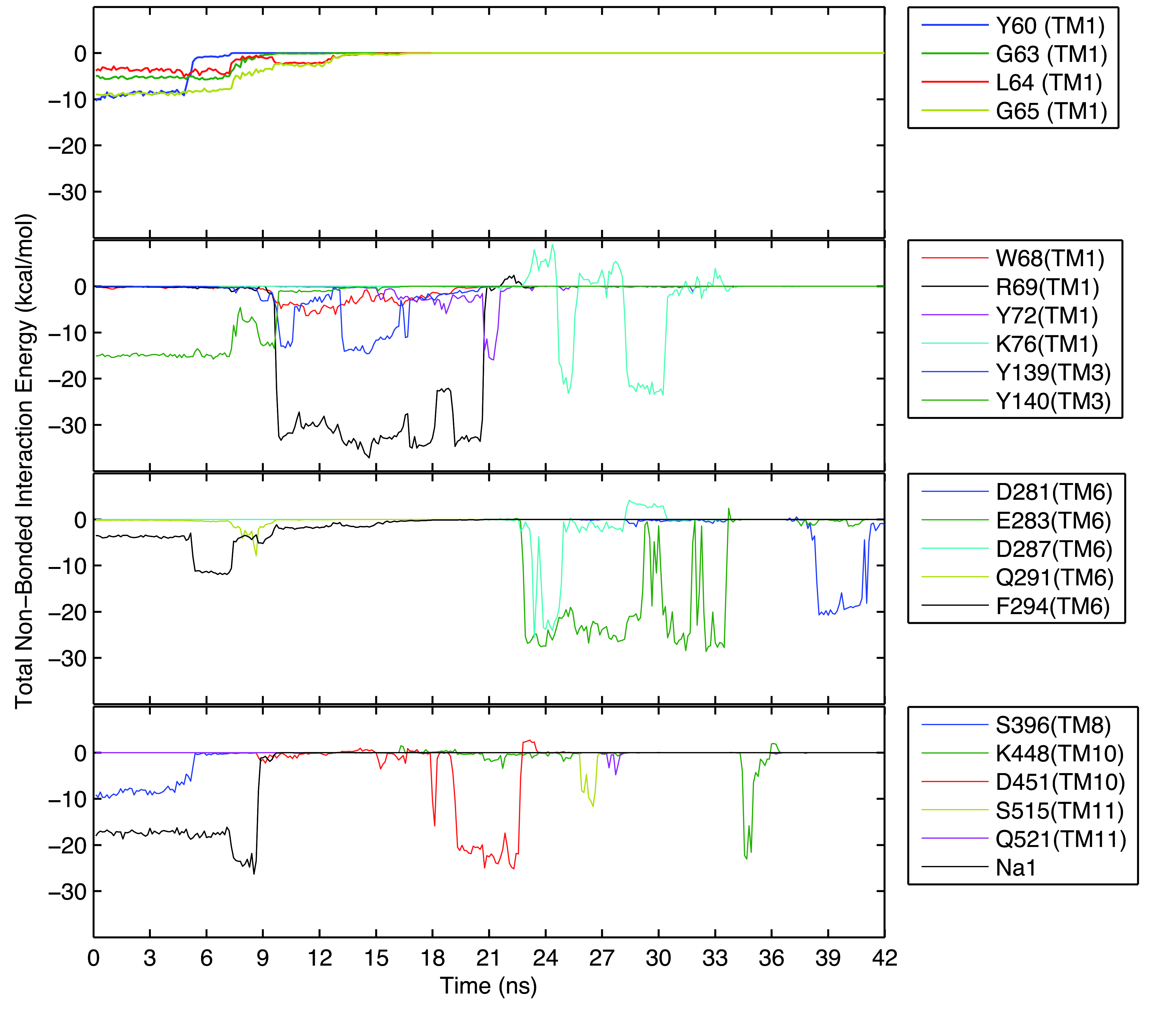

Supplement: Figure S2 — GABA dissociation using distance restraints. Non-bonded interaction energy profiles between GABA and residues interacted with during simulation. (TIF) [file pone.0039360.s002.tif]

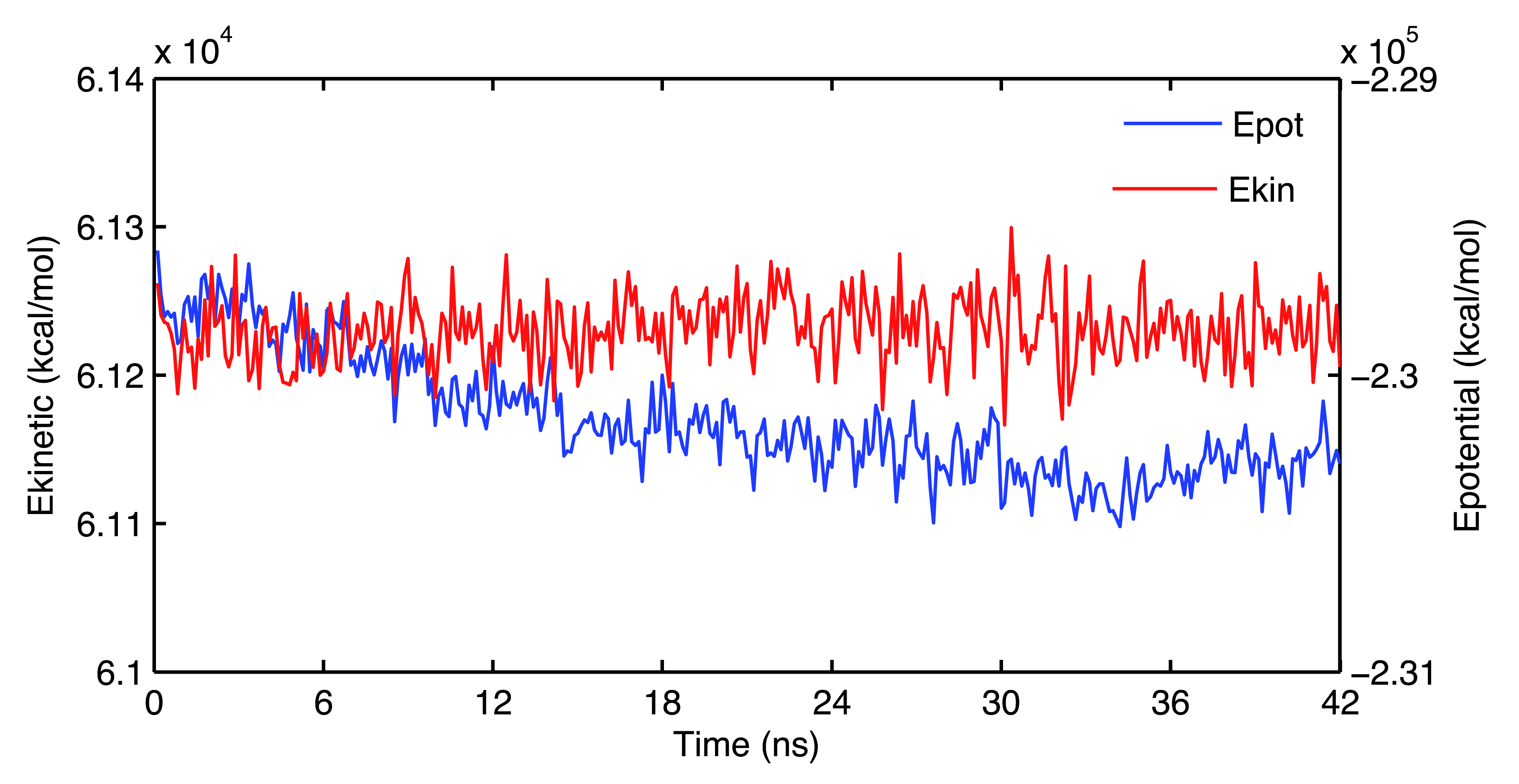

Supplement: Figure S3 — Evolvement of the kinetic and potential energy of the chemical system during dissociation of GABA. The kinetic energy (blue) is stable throughout the simulation, while the potential energy (red) is slightly decreasing between time 6 ns and 20 ns. During this time interval GABA is leaving the S1 site and is moving through the S2 site to the extracellular vestibule where it does not experience any further hindrance from the protein. The system is not in full equilibrium and hence is not the energies of the system. From the low-biased dissociation simulation with distance restraints. (TIF) [file pone.0039360.s003.tif]

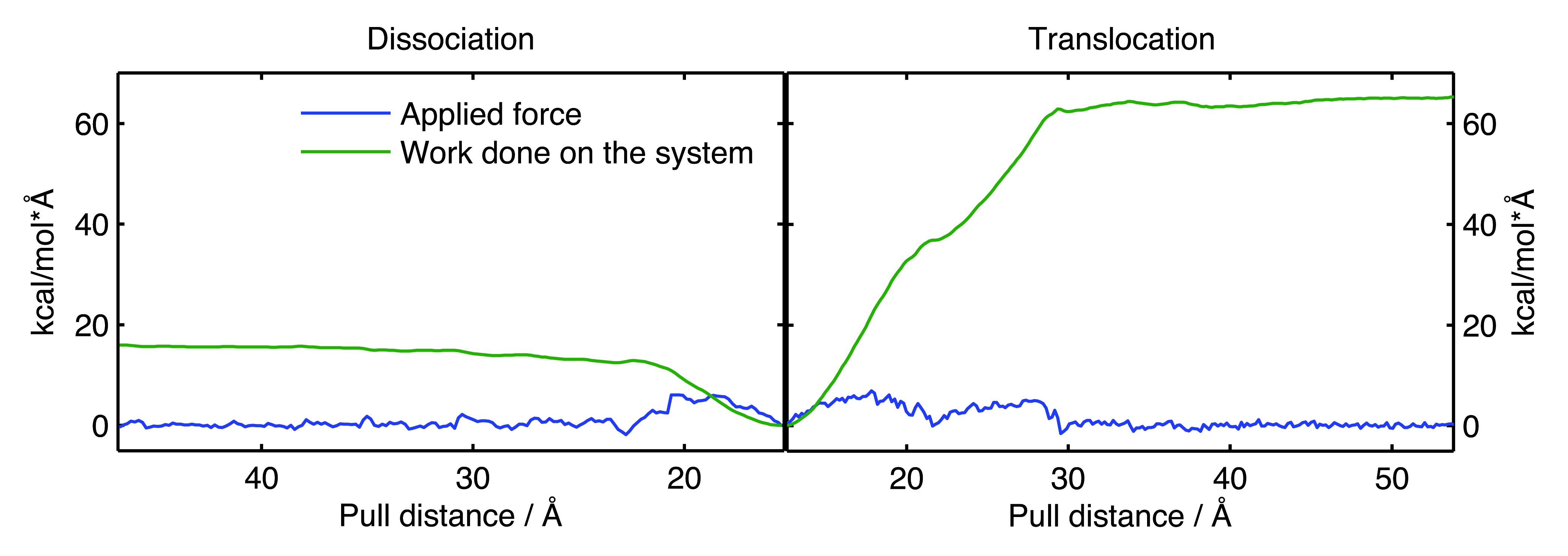

Supplement: Figure S4 — Applied force and work. Comparison of the applied force (blue curve) and the accumulated work done on the system (green curve) as a function of the distance traveled by GABA for the low-biased distance restrained dissociation (left-hand side) and translocation (right-hand side) simulations. The graphs reveal that the height of the potential energy barriers to cross are of equal magnitude in the two simulations, but the work necessary to translocate to the cytoplasm is three times the work to dissociate to the extracellular media. (TIF) [file pone.0039360.s004.tif]

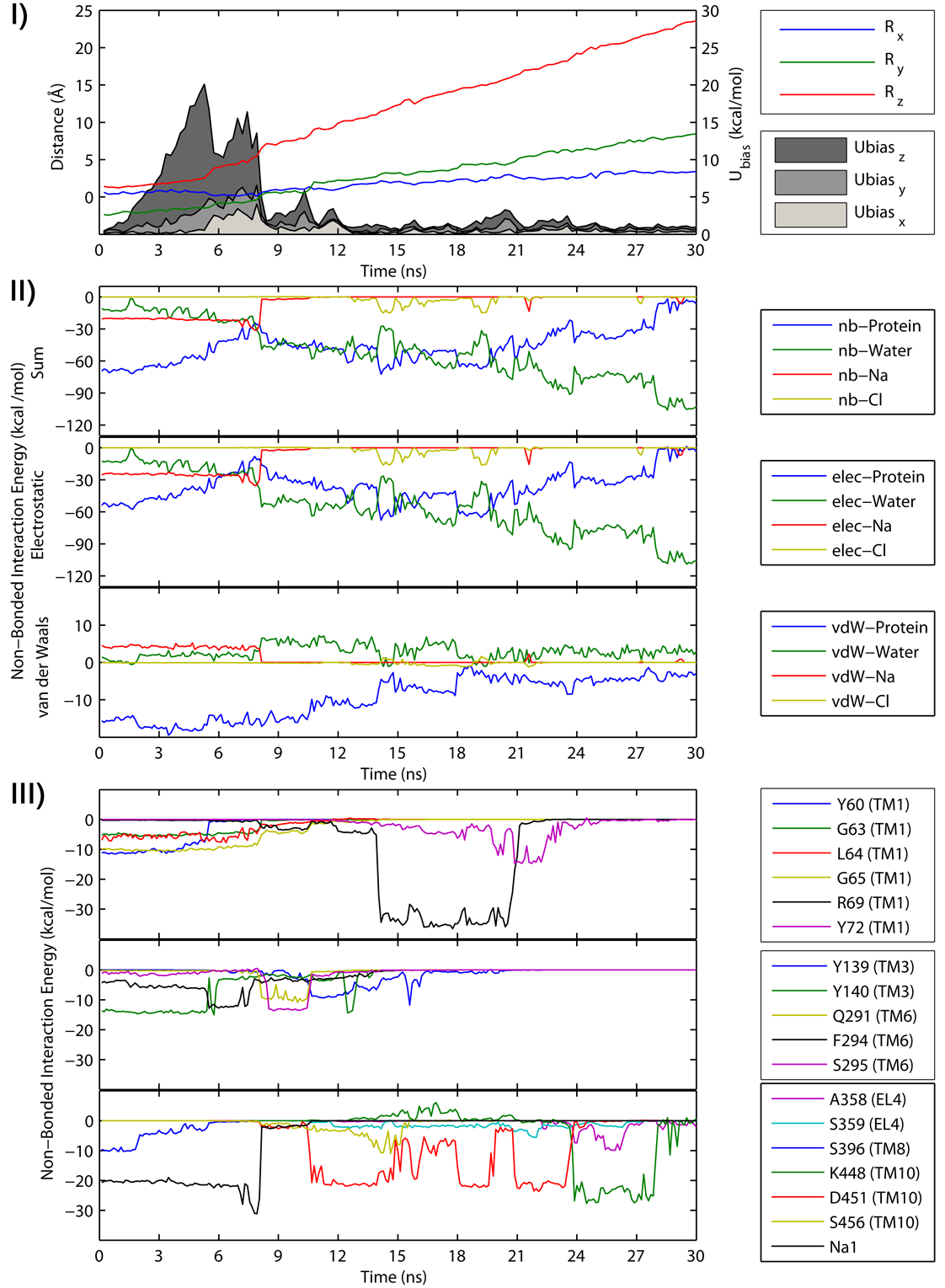

Supplement: Figure S5 — SMD profile for the dissociation of R- nipecotic acid. The individual figures from top to bottom show: the displacement (shown as lines) of the center of mass (COM) of R-nipecotic acid relative to the COM of the chemical system and the associated biasing potential energy profile (shown as stacked surfaces) (I); non-bonded interaction energy profiles between R-nipecotic acid and the protein, water, sodium- and chloride ions (II); non-bonded interaction energy profiles between R-nipecotic acid and residues interacted with during simulation (III). (TIF) [file pone.0039360.s005.tif]

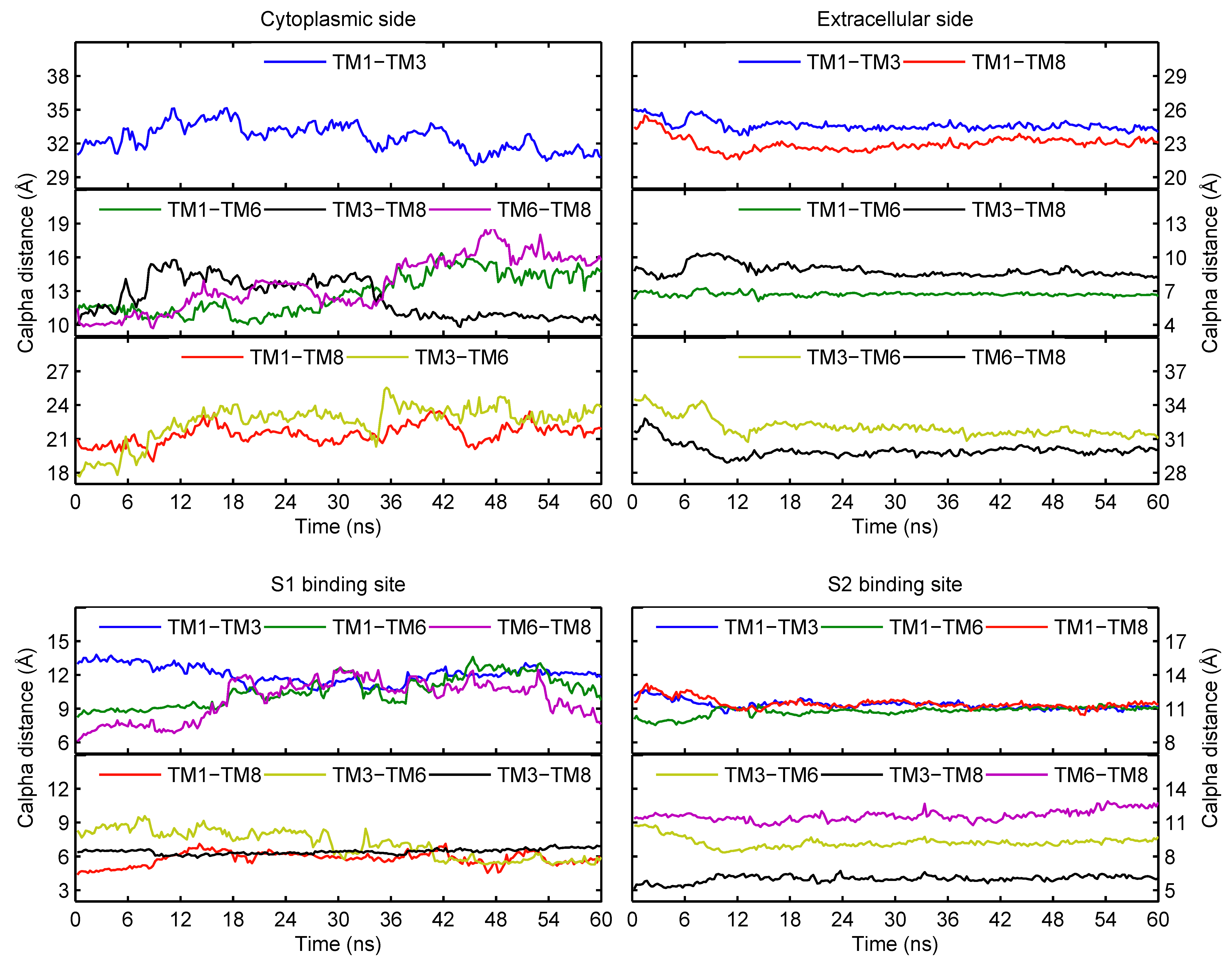

Supplement: Figure S6 — Intramolecular helix-helix distances from the GABA translocation simulation using displacement restraints. Graphs following the distances between the helices forming the S1 substrate binding site: TM1, TM3, TM6, and TM8. At the cytoplasmic gate the helices clearly move, and particularly the inner half of TM6 moves downwards and away from TM1. At the extracellular side the distances are relatively stable. (TIF) [file pone.0039360.s006.tif]

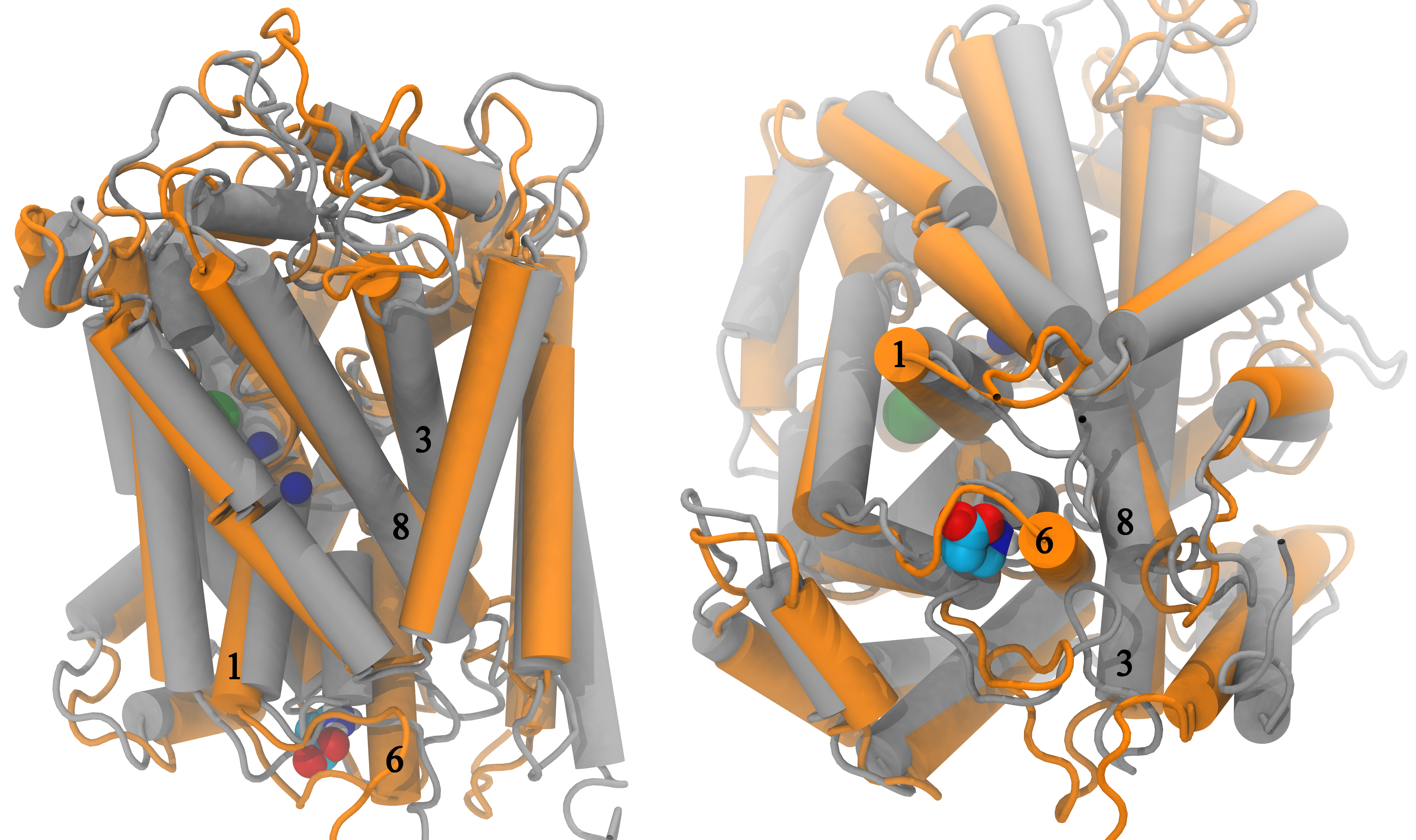

Supplement: Figure S7 — Structural changes associated with reorientation to the inward-open conformation. The initial outward-facing occluded protein conformation (grey) is compared to the resulting inward-open conformation (orange) resulting from the translocation of GABA to the cytoplasm (using displacement restraints). GABA (shown as light-blue spheres) is located at the cytoplasmic gate. The helices forming the S1 binding site, TM1, TM3, TM6, and TM8, are marked. The illustration is comparable to the illustration by Zhao et al. of LeuT [14]. (TIF) [file pone.0039360.s007.tif]

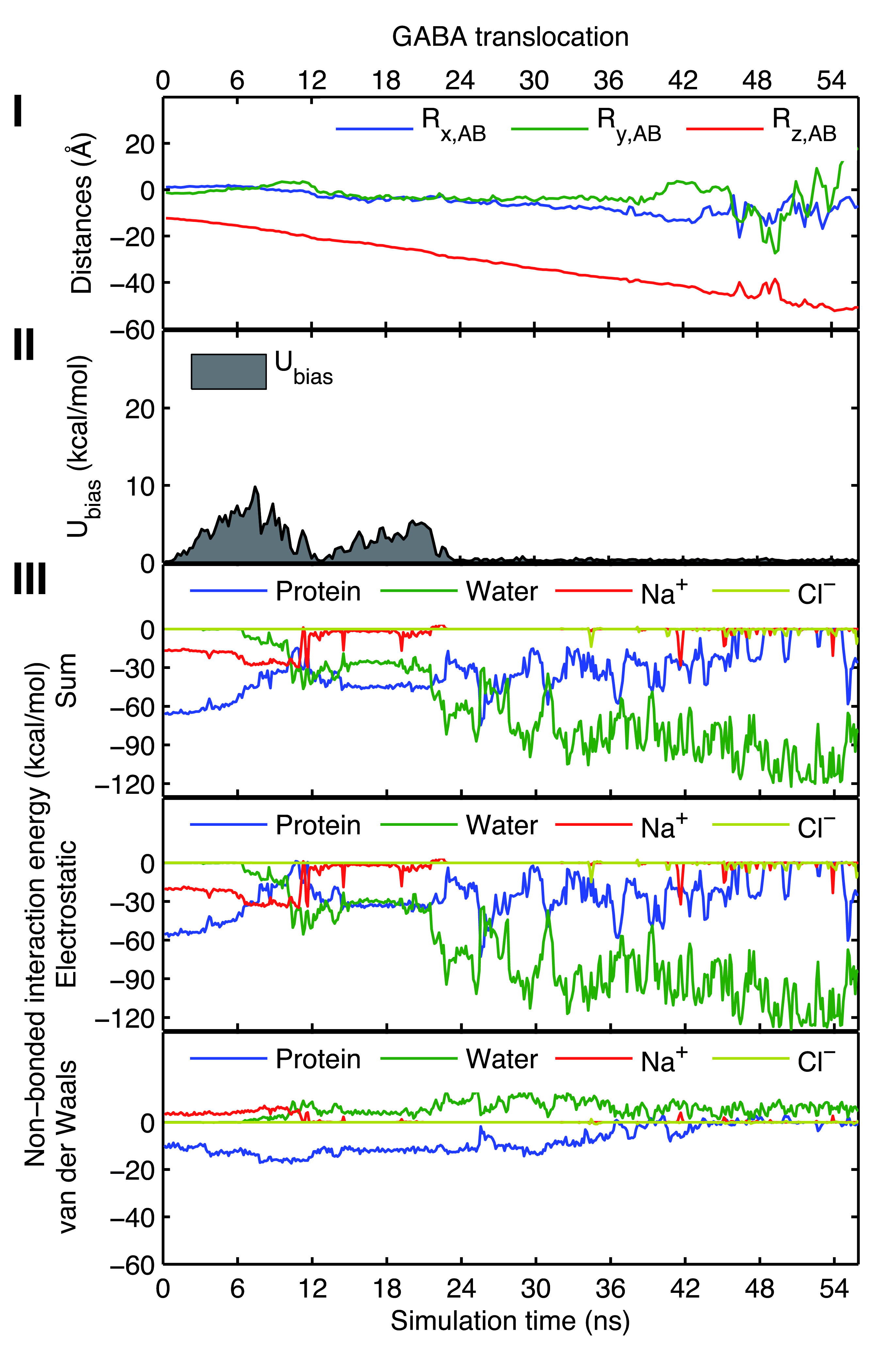

Supplement: Figure S8 — GABA translocation to the cytoplasm using distance restraints. The individual figures from top to bottom show: traces of the COM of GABA showing the distance of GABA relative to the extracellular entrance to the protein (I); the biasing potential energy profile (II); the non-bonded interaction energy profiles between GABA and the protein, water, sodium- and chloride ions, divided into the vdW and the electrostatic contributions as well as the sum of these (III). (TIF) [file pone.0039360.s008.tif]

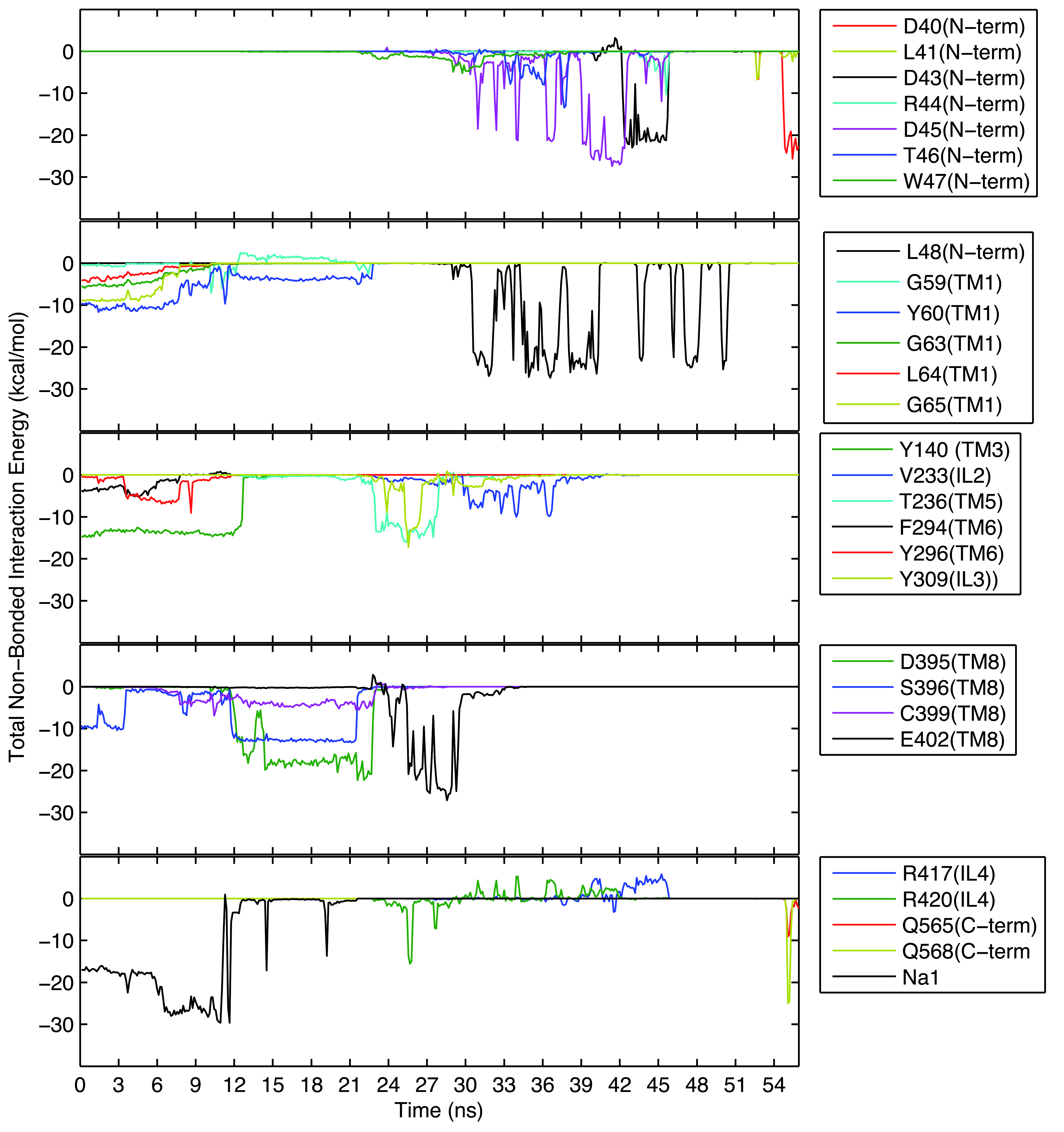

Supplement: Figure S9 — GABA translocation to the cytoplasm using distance restraints. Non-bonded interaction energy profiles between GABA and residues interacted with during simulation. (TIF) [file pone.0039360.s009.tif]

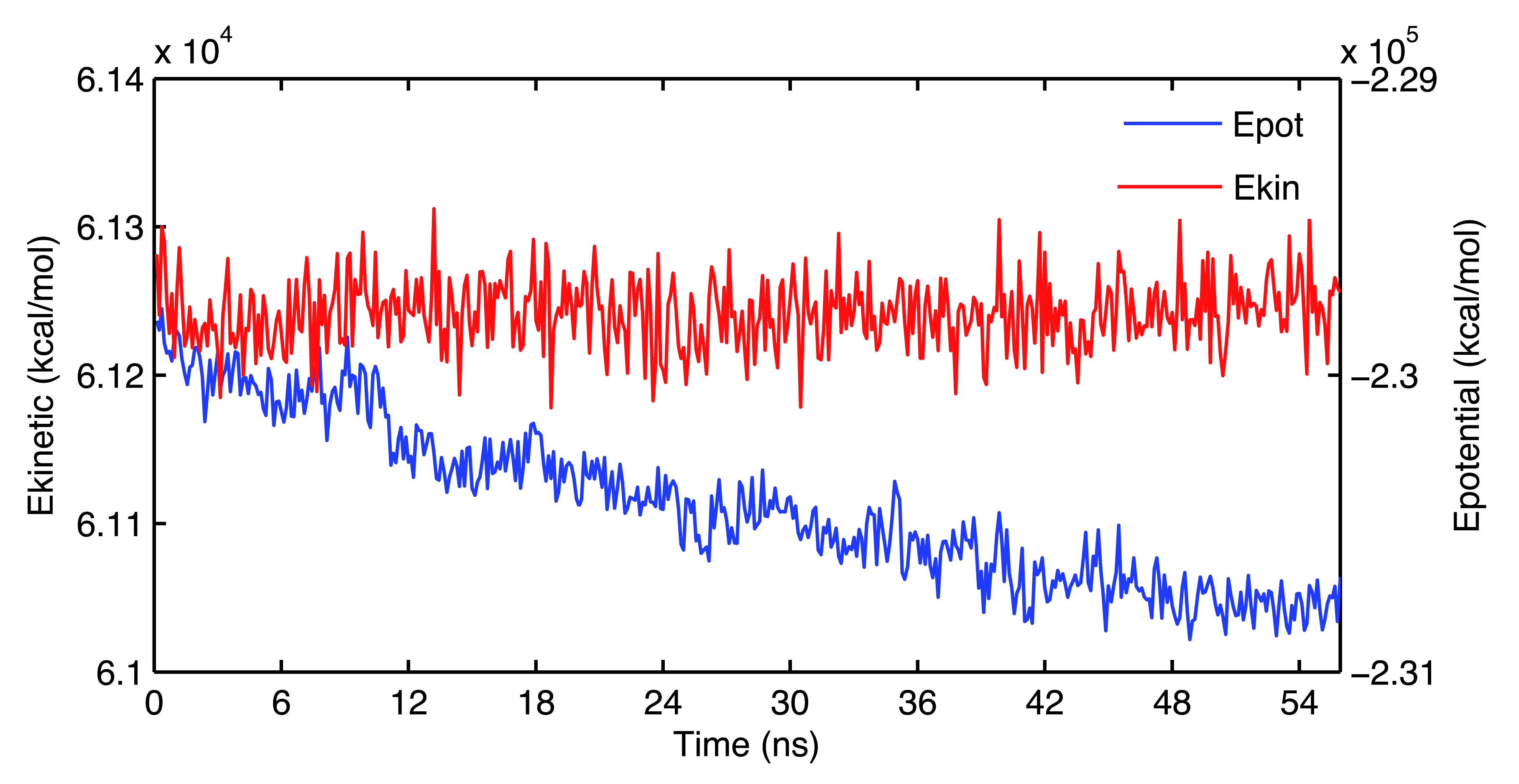

Supplement: Figure S10 — Evolvement of the kinetic and potential energy of the chemical system during translocation of GABA. The kinetic energy (blue) is stable throughout the simulation, while the potential energy (red) is slightly decreasing. The system is not in full equilibrium and hence is not the energies of the system. From the low-biased translocation simulation with distance restraints. (TIF) [file pone.0039360.s010.tif]
